# Conversion Surgery after Complete Response to Immune Checkpoint Inhibitor-Based Sequential Chemotherapy for Initially Unresectable Perihilar Cholangiocarcinoma: A Case Report

**DOI:** 10.70352/scrj.cr.25-0555

**Published:** 2025-12-23

**Authors:** Takahiro Tomino, Keishi Sugimachi, Tadatoshi Kakimoto, Takeshi Kurihara, Emi Onishi, Yutaka Koga, Kenichi Taguchi, Rie Sugimoto, Masaru Morita

**Affiliations:** 1 Department of Hepatobiliary and Pancreatic Surgery, NHO Kyushu Cancer Center, Fukuoka, Fukuoka, Japan; 2 Department of Gastrointestinal Surgery, Shin-Koga Hospital, Kurume, Fukuoka, Japan; 3 Department of Cancer Pathology, NHO Kyushu Cancer Center, Fukuoka, Fukuoka, Japan; 4 Department of Gastroenterology, NHO Kyushu Cancer Center, Fukuoka, Fukuoka, Japan; 5 Department of Gastroenterological Surgery, NHO Kyushu Cancer Center, Fukuoka, Fukuoka, Japan

**Keywords:** perihilar cholangiocarcinoma, pathological complete response, conversion surgery, GCS chemotherapy, GCD chemotherapy

## Abstract

**INTRODUCTION:**

Perihilar cholangiocarcinoma (PHCC) is often diagnosed at an advanced stage, and surgical resection is frequently precluded due to local progression, distant metastases, or insufficient liver remnant function. Although systemic chemotherapy has recently improved outcomes in unresectable biliary tract cancer, reports of conversion surgery after achieving pathological complete response (pCR) remain extremely rare, thus the role of salvage surgery remains unclear.

**CASE PRESENTATION:**

A woman in her 50s presented with abdominal distension and jaundice. Imaging revealed a bile duct stricture below the hepatic duct confluence with a 30-mm pericholedochal lymph node invading the hepatic arteries and portal vein bifurcation. Cytology confirmed adenocarcinoma, and she was diagnosed with unresectable PHCC (Bismuth type II, UICC T4N1M0, stage IIIC). First-line gemcitabine, cisplatin, and S-1 (GCS) chemotherapy was given for 8 courses, resulting in marked lymph node shrinkage, though vascular involvement persisted. Due to adverse events, the regimen was switched to gemcitabine, cisplatin, and durvalumab (GCD) for 7 courses, which maintained disease control. After 13 months of sequential chemotherapy, hepatic functional reserve was adequate, and left portal vein reconstruction with hepatic artery preservation was feasible. Conversion surgery consisting of right hemihepatectomy with portal vein resection/reconstruction and extrahepatic bile duct resection was performed. Pathology showed no residual tumor cells and extensive fibrosis with chronic inflammation, confirming a pCR. The patient has been receiving adjuvant S-1 and remains disease-free 4 months postoperatively.

**CONCLUSIONS:**

This case demonstrates that sequential chemotherapy—specifically GCS followed by GCD—can enable conversion surgery and possibly achieve pCR in initially unresectable PHCC. The case highlights the potential of sequential chemotherapy to expand curative options for this otherwise highly lethal disease.

## Abbreviations

BTC
: biliary tract cancer
CA19-9
: carbohydrate antigen 19-9
CEA
: carcinoembryonic antigen
CR
: complete response
FDG
: fluorodeoxyglucose
GC
: gemcitabine and cisplatin
GCD
: gemcitabine, cisplatin, and durvalumab
GCP
: gemcitabine, cisplatin, and pembrolizumab
GCS
: gemcitabine, cisplatin, and S-1
HH15
: heart-to-liver uptake ratio at 15 minutes
ICG
: indocyanine green
ICG-R15
: indocyanine green retention rate at 15 minutes
ICI
: immune checkpoint inhibitor
LHL15
: liver-to-heart uptake ratio at 15 minutes
MRCP
: magnetic resonance cholangiopancreatography
ORR
: objective response rate
pCR
: pathological complete response
PHCC
: perihilar cholangiocarcinoma
PV
: portal vein
RHA
: right hepatic artery
UICC
: Union for International Cancer Control

## INTRODUCTION

PHCC is an aggressive malignancy, often diagnosed at an advanced stage. Surgical resection is frequently not feasible due to distant metastasis, local progression, or insufficient future liver remnant function, causing the high risk of perioperative morbidity and mortality. Previous reports have shown that unresectable PHCC has a poor prognosis, with a 5-year survival rate of nearly zero in both Japan and the Western countries.^1–6)^

The treatment landscape for unresectable BTC has evolved significantly over the past 2 decades. The combination of GC, established as the standard 1st-line chemotherapy by the pivotal ABC-02 trial, marked a major milestone.^7)^ Subsequently, the KHBO1401 study demonstrated the efficacy of the triplet regimen comprising GCS, particularly in East Asian populations.^8)^ More recently, the incorporation of ICIs has transformed the therapeutic paradigm. The TOPAZ-1 trial showed a significant survival benefit with the addition of GCD chemotherapy.^9)^ Similarly, the combination of GCP is being investigated as a promising treatment strategy (KEYNOTE-966).^10)^ Recent advances in chemotherapy for unresectable BTC have led to increasing reports of successful conversion surgery following effective preoperative treatment, potentially improving prognosis.^11)^ However, there are few case reports of unresectable BTC that achieved pCR following conversion surgery.

Herein, we report a case of locally advanced PHCC that achieved pCR following conversion surgery following GCS and GCD chemotherapies. We also review previous case reports on pCR of conversion surgery following systemic chemotherapy for unresectable BTC (**Table 1**).

**Table 1:** Case reports on pCR of conversion surgery following systemic chemotherapy for unresectable biliary tract cancer

Author	Year	Age	Sex	Diagnosis	Reason for unresectability	Genetic alterations	First-line chemotherapy (cycles)	Reason for switching to 2nd-line chemotherapy	2nd-line chemotherapy (cycles)	Duration of chemotherapy (months)
Tran et al.	2015	67	M	Intrahepatic cholangiocarcinoma	Main portal invasion, extensive regional lymph nodes metastasis	Not described	GEMOX (4 cycles)	Progressive disease	GC (5 cycles)	Not described
Watanabe et al.	2017	70	F	Distal cholangiocarcinoma	Para-aortic lymph nodes metastasis	Not described	GS (32 cycles)	–	–	Not described
Adachi et al.	2019	69	F	Distal cholangiocarcinoma	Liver metastasis	Not described	GC (10 cycles)	–	–	7
Prieto et al.	2019	44	M	Gallbladder cancer	Liver and para-aortic lymph node metastases	HER2 mutation	GC (3 cycles)	Progressive disease	Capecitabine plus oxaliplatin and trastuzumab (8 cycles)	12
Sato et al.	2019	68	F	Ampullary carcinoma	Liver metastasis	Not described	GC (not described)	–	–	19
Oh et al.	2021	57	F	Perihilar cholangiocarcinoma	Bismuth type IV with PV and HA invasion, metastic lymph node along the common hepatic artery	Not described	GC (40 cycles)	–	–	30
Abudalou et al.	2021	47	M	Intrahepatic cholangiocarcinoma	Bilateral hepatic vein invasion, IVC invasion, para-aortic lymph nodes metastasis	TMB-high	GC + PTX/nab-PTX (5 cycles)	TMB-high	Pembrolizumab (5 cycles)	Not described
Miura et al.	2022	67	F	Gallbladder cancer	Pancreatic invasion, para-aortic lymph node metastasis	Not described	GC (22 cycles)	Repeated anemia	Gemcitabine (56 cycles)	48
Orlandi et al.	2024	62	M	Gallbladder cancer	Duodenal invasion	None	GCD (8 cycles) → durvalumab (not described)	–	–	17
Shimamaki et al.	2024	79	F	Intrahepatic cholangiocarcinoma	IVC invasion, PV invasion, HA invasion, regional lymph nodes metastasis	Not described	GC (4 cycles)	–	–	Not described
Yasui et al.	2025	70	M	Perihilar cholangiocarcinoma	Bismuth type II with para-aortic lymph nodes metastasis	Not described	GCD (8 cycles)	–	–	Not described
Fukuda et al.	2025	64	M	Intrahepatic cholangiocarcinoma	Para-aortic lymph nodes metastasis	Lynch syndrome with mismatch repair deficiency (dMMR: loss of MLH1/PMS2)	GC (4 cycles)	Progressive disease	GCD (10 cycles)	12
Inokawa et al.	2025	69	M	Perihilar cholangiocarcinoma	Bismuth type IV with PV and HA invasion	MSI-high and TMB-high	GC (5 cycles)	Slight tumor progression ranging the status of SD	Pembrolizumab (24 cycles)	23
Morita et al.	2025	52	F	Intrahepatic cholangiocarcinoma	Regional lymph nodes metastasis	BRCA2	GCS (7 cycles)	–	–	Not described
Present case	2025	54	F	Perihilar cholangiocarcinoma	Bismuth type II with PV and HA invasion of pericholedochal lymph node	Not tested	GCS (8 cycles)	Intolerable dysgeusia and stomatitis	GCD (7 cycles)	11

BRCA2, breast cancer susceptibility gene 2; dMMR, deficient mismatch repair; F, female; GC, gemcitabine and cisplatin; GCD, gemcitabine, cisplatin, and durvalumab; GCS, gemcitabine, cisplatin, and S-1; GEMOX, gemcitabine and oxaliplatin; GS, gemcitabine and S-1; HA, hepatic artery; HER2, human epidermal growth factor receptor 2; IVC, inferior vena cava; M, male; MLH1, mutL homolog 1; MSI-high, microsatellite instability-high; pCR, pathological complete response; PMS2, postmeiotic segregation increased 2; PTX, paclitaxel; PV, portal vein; SD, stable disease; TMB, tumor mutational burden

## CASE PRESENTATION

**Fig. 1:**
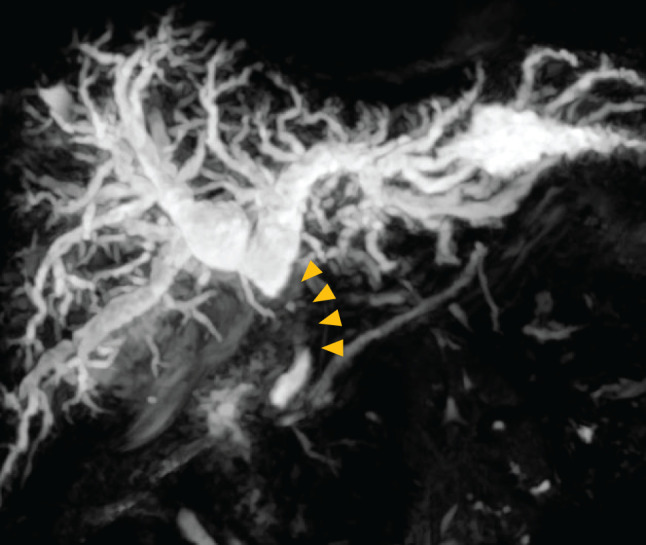
Findings of MRCP at initial diagnosis. Strictures of the hepatic and common bile ducts are observed (yellow arrowhead). MRCP, magnetic resonance cholangiopancreatography

**Fig. 2:**
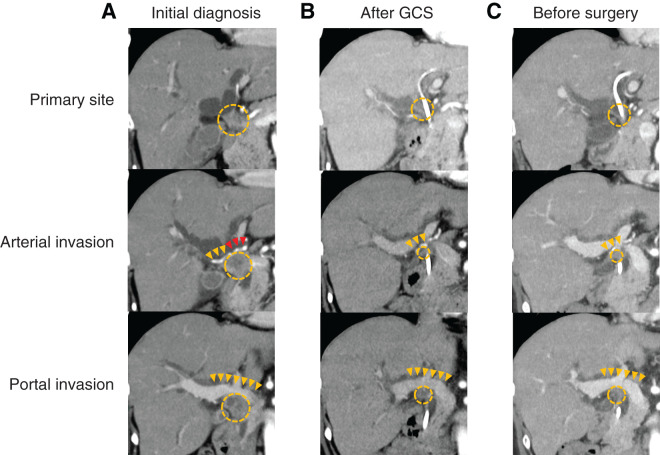
Findings of contrast-enhanced CT at initial diagnosis (**A**), after GCS chemotherapy (**B**) and before surgery (**C**). (**A**) A bile duct stricture extending from just below the hepatic duct confluence to the upper border of the pancreas was noted (dotted circle in the upper panel). A 30-mm pericholedochal lymph node metastasis was observed (dotted circle in the middle panel), along with invasion of the right hepatic artery (yellow arrowhead) and contact of left hepatic artery (red arrowhead) by the lymph node. Invasion of the main portal vein and its right branch by an enlarged pericholedochal lymph node (dotted circle in the lower panel) was observed (yellow arrowhead). (**B**) The stricture of the perihilar bile duct showed marked improvement (dotted circle in the upper panel). Marked shrinkage of the metastatic pericholedochal lymph node was observed (dotted circle in the middle and lower panel). The stenosis of the right hepatic artery and PV was persistent (yellow arrowhead). The contact between the metastatic lymph node and the hepatic artery disappeared. (**C**) The tumor remained shrunken (dotted circle in the upper panel). The size of the metastatic lymph node before surgery was persistent after GCS chemotherapy (dotted circle in the middle and lower panel). The deformation of the right hepatic artery and portal vein persisted (yellow arrowhead). GCS, gemcitabine, cisplatin, and S-1; PV, portal vein

**Fig. 3:**
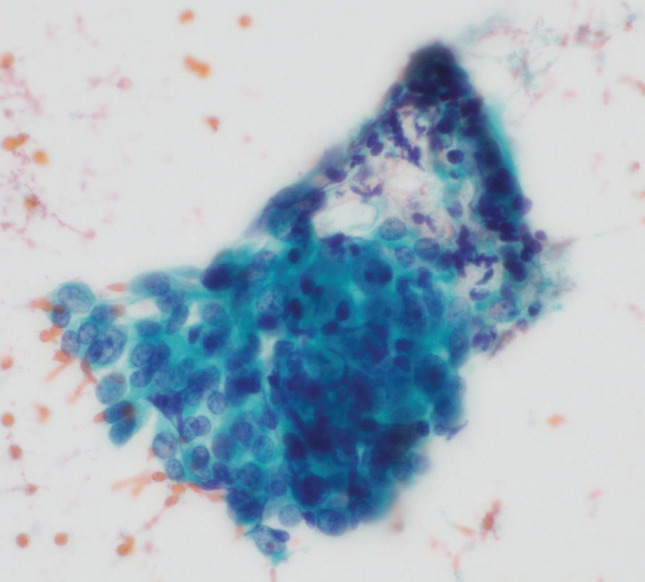
Pathological finding of biliary brush cytology. Biliary brush cytology revealed atypical cells showing irregular overlapping, nuclear disarray, and uneven contours of the cell clusters, leading to a diagnosis of Class V (adenocarcinoma).

**Fig. 4:**
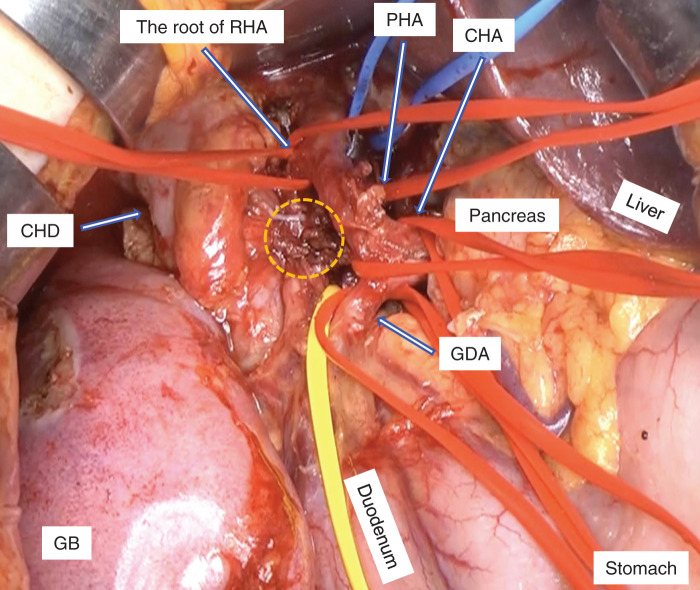
Intraoperative finding. The root of RHA was indurated due to post-chemotherapy change and the metastatic pericholedochal lymph node (dotted circle) had decreased in size. The root of RHA was isolated and taped. CHA, common hepatic artery; CHD, common hepatic duct; GB, gallbladder; GDA, gastroduodenal artery; PHA, proper hepatic artery; RHA, right hepatic artery

**Fig. 5:**
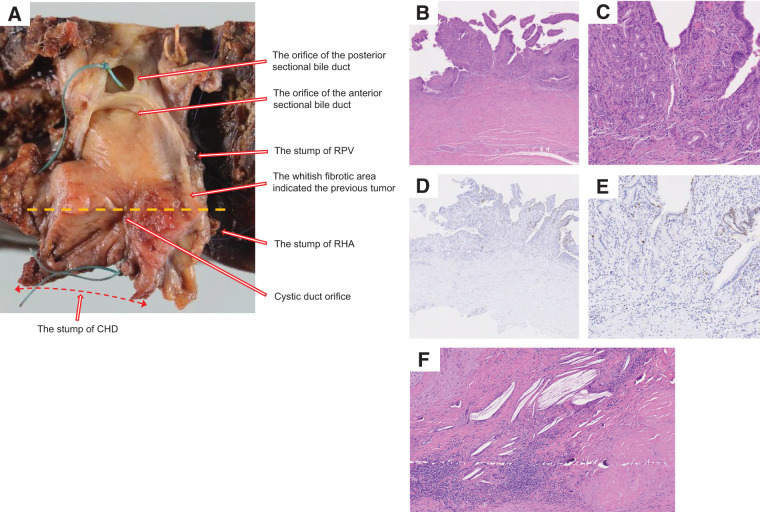
Gross inspection and pathological findings of resected specimen. (**A**) A retraction of the bile duct wall was observed from just below the bifurcation of the right and left hepatic ducts to just above the distal end of the common bile duct (double red arrow). The tumor section was indicated by a dotted line. (**B**, **C**) Microscopic views of tumor section at 100× and 200× magnification in hematoxylin and eosin staining revealed no residual tumor cells. The bile duct exhibited fibrosis accompanied by chronic active inflammatory changes. (**D**, **E**) Microscopic views of tumor section at 100× and 200× magnification in immunohistochemical staining for Ki67 revealed no increase in the Ki67 labeling index. (**F**) Microscopic examination of a metastatic lymph node at ×100 magnification with hematoxylin and eosin staining revealed that the adipose tissue outside the bile duct wall had been replaced by fibrous connective tissue, accompanied by lymphocytic aggregation, calcification, cholesterol crystals, and a foreign-body reaction. CHD, common hepatic duct; RHA, right hepatic artery; RPV, right portal vein

A woman in her 50s with no significant past medical history presented to a local clinic with abdominal distension and jaundice. MRCP revealed a bile duct stricture below the hepatic duct confluence (**Fig. 1**). Contrast-enhanced CT showed a bile duct stricture extending from just below the hepatic duct confluence to the upper border of the pancreas, accompanied by an enlarged pericholedochal lymph node (maximum diameter: 30 mm) that was invading the right and left hepatic arteries and the portal vein bifurcation (**Fig. 2A**). The pericholedochal lymph node showed contrast enhancement on contrast-enhanced CT. Based on the CT findings, lymph nodes larger than 1 cm with a round morphology and contrast enhancement were considered metastatic on imaging. Evaluation by endoscopic ultrasonography or PET-CT was not performed. An endoscopic retrograde cholangiopancreatography with biliary brush cytology revealed atypical cells showing irregular overlapping, nuclear disarray, and an uneven contour of the cell clusters, leading to a diagnosis of Class V (adenocarcinoma) (**Fig. 3**), and the patient was diagnosed with unresectable perihilar cholangiocarcinoma (Bpd, Bismuth type II, UICC clinical T4N1M0, stage IIIC). Bile duct biopsy and intraductal ultrasonography were not performed at diagnosis. Serum tumor markers CEA and CA19-9 were within the normal range. In this case, the tumor at the initial presentation appeared as a bulky mass contiguous with metastatic lymph nodes, extending from the superior border of the pancreas to the hepatic hilum. It further invaded the proper hepatic artery and the portal vein. Therefore, we determined that the disease was locally advanced and technically unresectable, and curative resection was deemed infeasible. Accordingly, GCS chemotherapy was initiated. After 8 courses, the patient developed dysgeusia and stomatitis, and the regimen was switched to 2nd-line chemotherapy. A contrast-enhanced CT revealed improvement of the perihilar bile duct stricture and marked shrinkage of the metastatic pericholedochal lymph node (maximum diameter: 10 mm; **Fig. 2B**). The contact between the metastatic lymph node and the left hepatic artery was improved while the stenosis of the RHA and PV was persistent (**Fig. 2B**). The 2nd-line chemotherapy regimen selected was GCD therapy, and 7 courses were administered. A contrast-enhanced CT revealed persistent involvement of the RHA and PV, with sustained shrinkage of the tumor and lymph node (maximum diameter: 11 mm; **Fig. 2C**). She was referred to our institution to consider the indication of conversion surgery. ICG test showed preserved hepatic function (ICG-R15: 4.0%). CT evaluation estimated that future liver remnant volume after right hemihepatectomy was 37.3%. Asialoscintigraphy demonstrated an HH15 of 0.483 and an LHL15 of 0.954. These values indicated that the future remnant liver had sufficient functional reserve for right hemihepatectomy. PET-CT demonstrated slight FDG uptake at the biliary stricture, probably related to inflammation from the biliary stent, but no abnormal uptake was observed in the metastatic lymph nodes or elsewhere. Based on detailed preoperative CT evaluation, we planned conversion surgery 13 months after initiation of chemotherapy, consisting of right hemihepatectomy with PV resection/reconstruction and extrahepatic bile duct resection/reconstruction. During surgery, intraoperative ultrasound confirmed that the biliary stricture was confined to the suprapancreatic portion. The root of RHA surrounded by neurovascular stroma was isolated and taped. The metastatic pericholedochal lymph node had decreased in size, and the area around the root of RHA was indurated due to post-chemotherapy changes (**Fig. 4**). Intraoperative frozen section of adjacent pericholedochal lymph node and neurovascular stroma around the RHA showed no malignancy. The common bile duct was taped and transected at the upper pancreatic margin. Dense adhesion between the right portal branch and bile duct led to the decision for wedge resection of the PV. The main portal trunk and left portal vein were then clamped, and wedge resection of the PV was performed. Right hemihepatectomy was performed, and the single-hole left hepatic duct was reconstructed via hepaticojejunostomy. According to the preoperative examination, we assumed that tumor invasion into the left hepatic duct was minimal. Therefore, a right hemihepatectomy with preservation of the left caudate (Spiegel) lobe was performed to maximize the future liver remnant. If the intraoperative frozen section of the left hepatic duct had been positive, we planned to perform additional resection of the caudate lobe; however, since the margin was negative, the caudate lobe was preserved. Gross inspection of the resected specimen showed whitish, dense, fibrotic scar tissue (**Fig. 5A**). Final pathological examination revealed no residual tumor cells and fibrosis with chronic active inflammation, indicating a pathological complete response (**Fig. 5B** and **5C**). Immunohistochemical staining for Ki67 revealed no increase in the Ki67 labeling index (**Fig. 5D** and **5E**). Microscopic examination of a metastatic lymph node with hematoxylin and eosin staining revealed that the adipose tissue outside the bile duct wall had been replaced by fibrous connective tissue, accompanied by lymphocytic aggregation, calcification, cholesterol crystals, and a foreign-body reaction. These findings suggested that the lesion might represent a previously metastatic lymph node that had regressed as a result of treatment effect (**Fig. 5F**). The patient started adjuvant S-1 chemotherapy. Four months after surgery, the patient remains alive without recurrence.

## DISCUSSION

We herein reported a case in which conversion surgery was safely performed after sequential chemotherapy, including an ICI for an initially unresectable PHCC, and pCR was confirmed by pathological examination. Achieved pCR cases of conversion surgery following systemic chemotherapy in unresectable BTC have been rarely reported. We conducted a systematic review of the English-language literature in PubMed using the keywords “neoadjuvant chemotherapy,” “preoperative chemotherapy,” “conversion surgery,” “cholangiocarcinoma,” and “complete response.” We identified 14 reported cases, which, together with the present case, are summarized in **Table 1** as examples of pCR in initially unresectable BTC after conversion surgery following systemic chemotherapy. The median patient age was 67 years. Primary diagnoses included PHCC (n = 4), gallbladder cancer (n = 3), distal cholangiocarcinoma (n = 2), ampullary carcinoma (n = 1), and intrahepatic cholangiocarcinoma (n = 5). Reasons for initial unresectability varied, including distant metastases, major vascular invasion, regional lymph node metastasis, or locally advanced disease. Of the 15 cases, a doublet regimen was administered in 9 cases and a triplet regimen in 6 cases. The median duration of chemotherapy was 21 months in the doublet regimen group and 12 months in the triplet regimen group. Second-line sequential therapy was administered in 7 cases, 4 of which included ICIs. All 4 cases, including the present 1, were reported after 2021. ICI-based sequential chemotherapy was administered in 4 cases. The median duration of chemotherapy was 12 months in the ICI-based group and 18 months in the non-ICI-based group. These findings suggest that a triplet regimen and sequential chemotherapy incorporating an ICI regimen may be a promising strategy for patients considered for conversion surgery, facilitating earlier achievement of pCR and highlighting the potential of such regimens in enabling curative-intent surgery.

Triplet regimens such as GCS, GCD, and GCP therapies have shown promise in tumor downstaging for unresectable BTC. In the KHBO1401 study, the ORR was significantly higher in the GCS group (41.5%) compared with the GC group (15.0%), with CR rates of 3% and 1%, respectively. The median duration of response was 5.95 months in the GCS group versus 5.49 months in the GC group. Notably, this study also evaluated the conversion rate, which was 0% in the GC group and 3% in the GCS group. Furthermore, the waterfall plot demonstrated that GCS induced substantial tumor shrinkage, highlighting its potential utility as a conversion-oriented regimen.^8)^ In the present case as well, the enlarged lymph node showed marked shrinkage with GCS chemotherapy, suggesting the potent tumor-reducing effect of the GCS regimen. In the TOPAZ-1 trial, the ORR was 18.7% in the GC plus placebo group and 26.7% in the GCD group. The CR rates were 0.6% and 2.1%, respectively. The median duration of response was 6.2 months for GC plus placebo and 6.4 months for GCD. The KEYNOTE-966 trial reported an ORR of 29% in both the GC plus placebo and GCP groups, with CR rates of 1% and 2%, respectively. The median duration of response was 6.9 months in the GC plus placebo group and 9.7 months in the GCP group. When comparing these 3 treatments, the ICI regimens tended to show a longer median duration of response. In addition, GCD was switched to durvalumab monotherapy as maintenance therapy from the 9th course onward, which was associated with fewer adverse events. Therefore, in cases requiring preoperative preparation such as portal vein embolization for PHCC, the ICI regimens may be potentially more suitable as a preoperative chemotherapy prior to surgery than non-ICI regimens.

It should be noted that these randomized controlled trials included not only PHCC but also other types of BTCs. A retrospective study by Takahashi et al. investigated preoperative chemotherapy in 31 patients with advanced PHCC only. Regimens included GCS (87.2%), GC (6.4%), and GCD (6.4%), with a median of 5 treatment cycles. Notably, a pCR was achieved in 12.9% of cases.^12)^ Reports of pCR following sequential chemotherapy with multiple regimens in unresectable PHCC are limited. Sequential chemotherapy, which combines the advantages of the strong shrinkage effect of GCS and the long response period of ICI, may be a promising treatment for BTC, especially PHCC, that allows for conversion surgery.

The definition of unreconstructable PHCC has not yet been clearly established. Although few reports have addressed the definition of unresectable PHCC, the present study defined unresectability in upper biliary tract cancer as unreconstructable bile duct invasion, insufficient future remnant liver volume, direct invasion of adjacent organs such as the pancreas, unreconstructable major vascular invasion, extranodal extension of regional lymph nodes, distant metastasis, and metastasis to non-regional lymph nodes.^13)^ Cases with distant metastasis, unreconstructable vascular invasion, or insufficient remnant liver volume are generally considered unresectable; however, the definition of unresectable PHCC has not been strictly established in current guidelines, and the development of a consensus is warranted.

At present, there are few data available regarding the definition of unresectable PHCC, nor on the optimal regimen or duration of treatment. It also remains unclear which factors define unresectable, and whether 2nd-line treatment should be administered after 1st-line failure or sequential treatment should be performed as planned. Further accumulation of cases is warranted to establish a clear definition of unresectable PHCC and to determine optimal treatment strategies that achieve higher conversion and pCR rates.

## CONCLUSIONS

This case demonstrated that sequential chemotherapy with multiple regimens, specifically GCS followed by GCD, could enable conversion surgery and achieve pCR in patients with initially unresectable PHCC. Although rare, such cases highlight the growing potential of sequential chemotherapy to achieve pCR in unresectable PHCC.

## DECLARATIONS

### Use of artificial intelligence tools

The authors used ChatGPT (developed by OpenAI) to assist in improving the English grammar and clarity of the manuscript. The final content was reviewed and approved by all authors.

### Availability of data and materials

The datasets used and/or analyzed during the current study are available from the corresponding author on reasonable request.
